# Modelling the Consequences of Domestication‐Introgression in Wild Populations Using Genetic Markers Under Varying Degrees of Selection

**DOI:** 10.1111/eva.70140

**Published:** 2025-09-19

**Authors:** K. A. Glover, M. Castellani, M. Heino, F. Besnier

**Affiliations:** ^1^ Institute of Marine Research Bergen Norway; ^2^ Department of Mechanical Engineering University of Birmingham Birmingham UK; ^3^ Department of Biological Sciences University of Bergen Bergen Norway

**Keywords:** admixture, aquaculture, de‐domestication, eco‐genetic modelling, farmed escapees, genetic interactions

## Abstract

Introgression of domesticated genomes influences the evolutionary trajectory of wild populations. Genetic markers are used to quantify admixture in wild populations subjected to introgression from non‐native conspecifics. However, markers can be under direct and indirect selection which may influence admixture estimates and quantification of fitness consequences thereafter. We expanded the Atlantic salmon eco‐genetic model IBSEM to compute individual fish phenotype and domestication admixture using markers under variable strengths of selection. Following 50 years of 5%–25% domesticated conspecifics on the spawning grounds, the recipient wild population showed an increase in adult size at age and a decline in adult abundance, both of which scaled with the degree of intrusion. In the following 50‐year recovery period without further escapees, traits started to but did not completely revert to pre‐impact levels. Neutral and weakly selected markers gave higher admixture estimates than markers under stronger degrees of selection. The disparity increased during the recovery period where neutral markers and their corresponding admixture estimates “lingered” in the wild population, whereas admixture based on markers under selection declined as the population recovered. During the recovery period, the strength in the relationship between individual fish admixture and size at age was also eroded when computed using neutral markers, but less so for the markers under selection. Collectively, these observations illustrate how markers under selection mirror the fitness and phenotypic changes in the population, while neutral markers reflect demographic history and can therefore not be uncritically used to infer fitness consequences. Our results also suggest that management guidelines used in Norway and some other countries, setting 10% domesticated escapees in a river and/or 10% domestication admixture in wild populations as the limit for a “large” impact, will provide a high level of protection for wild salmon populations.

## 1 Introduction

The continued expansion of the human population is putting a strain on the planet's resources. Consequently, it has never been so important as now to provide robust scientific advice on the potential impacts and sustainability of human activities. Aquaculture is one of the fastest growing food‐production sectors and is practiced in all regions of the globe (Bostock et al. 2010; Naylor et al. 2021). However, many forms of aquaculture challenge the natural environment (Naylor et al. 2000; Cao et al. 2007; Edwards 2015; Taranger et al. 2015). Within the Atlantic salmon ( *Salmo salar* ) aquaculture industry, genetic interactions between domesticated escapees and wild conspecifics represent one of the most persistent challenges to environmental sustainability (Taranger et al. 2015), with nearly five decades of intensive research and debate (Hindar et al. 1991; Naylor et al. 2005; Glover et al. 2017). Therefore, Atlantic salmon is an important model system of genetic interactions between aquaculture escapees and wild populations.

Domestication is increasingly a prerequisite for viable aquaculture, as evidenced by the increasing number of finfish species being domesticated (Teletchea and Fontaine 2014). It involves genetic changes rendering the target population more productive in the human‐controlled environment (Price 1984, 1999). However, breeding programs do not only change traits under directional selection, as the general effects of domestication often change traits that are not intentionally selected for, such as susceptibility to predation (Solberg et al. 2020) and phenology (Skaala et al. 2019). Atlantic salmon were first taken into breeding programs in the early 1970's (T. Gjedrem 2000, 2010), and after 10 or more generations display a range of genetic differences to wild Atlantic salmon (Glover et al. 2017), with demographic consequences including lower survival in the natural environment (McGinnity et al. 1997; Fleming et al. 2000; Skaala et al. 2012, 2019; Wacker et al. 2021). These changes are a concern because domesticated escapees have been observed on the spawning grounds of many rivers for several decades (Morris et al. 2008; Diserud et al. 2019; Glover et al. 2019), and introgression has been documented in multiple populations in several countries such as Norway (Glover et al. 2013; Karlsson et al. 2016), Canada (Wringe et al. 2018; Bradbury et al. 2022), Northern Ireland (Crozier 1993, 2000), Ireland (Clifford et al. 1998a, 1998b) and Scotland (Gilbey et al. 2018). Introgression has also been documented to result in phenotypic (Bolstad et al. 2017, 2021; Besnier et al. 2022) and phenological changes in native populations (Besnier et al. 2022).

To quantify introgression and study the fitness consequences of gene flow from domesticated to wild populations, molecular genetic markers are being used to identify domesticated, wild, and admixed individuals. In Norway, introgression has been quantified in ~250 Atlantic salmon populations (Glover et al. 2013; Karlsson et al. 2016; Diserud et al. 2022), thus providing a globally unprecedented picture of gene flow from any domesticated to wild conspecific. This was achieved using genome‐mined SNPs that are statistically informative for multiple domesticated Atlantic salmon strains and wild salmon populations (Karlsson et al. 2011). Similar work to find aquaculture‐wild informative/diagnostic SNPs for salmon has also been conducted in North America (Liu et al. 2017; Wringe et al. 2019), and for a single Norwegian population that is being comprehensively dissected to look at the effects of domestication introgression (Besnier et al. 2022). However, it is likely that some of the markers that are statistically informative or potentially diagnostic for domesticated vs. wild populations are under some degree of selection (Lopez, Benestan, et al. 2019; Lopez, Linderoth, et al. 2019; Naval‐Sanchez et al. 2020). Markers under selection may bias estimates of introgression as they are purged from the recipient population at a higher rate than neutral markers (Edmands 2007), unless selection is weak or highly sporadic, or populations are small and genetic drift leads to near or complete fixation of maladaptive alleles before they can be removed by selection (Ellstrand et al. 1999). Therefore, it follows that estimates of introgression and studies of fitness consequences may be influenced by such mechanisms.

A better understanding of how genetic markers under differing degrees of selection may affect estimates of introgression, and thereafter, estimates of phenotypic, demographic, and phenological changes in wild populations is needed. The Individual‐Based Atlantic Salmon Population Model IBSEM (Castellani et al. 2015) has previously provided population‐level insights into the expected demographic and phenotypic changes in wild salmon populations following spawning intrusion of domesticated conspecifics (Castellani et al. 2018; Sylvester et al. 2019). In the present study, we made further developments to IBSEM to permit simultaneous quantification of phenotypic changes and admixture in individual fish, the latter computed according to either an exact ancestry or genetic markers under neutral and low (hereon collectively referred to as weak or low), middle, and high degrees of selection. Thereafter, we used these estimates to investigate the effect of introgression on population abundance, the relationship between adult size at age and individual admixture, and how this varied with the types of markers used to compute admixture. Scenarios were modeled on a time scale and with a level of spawning intrusion that overlaps with the situation(s) observed in a number of salmon populations in the wild, thus providing the first modeled outputs that are of direct relevance for the management authorities.

## 2 Methods

### 2.1 Overall Study Design

The study was divided into two main parts. First, we extended the model IBSEM to enable it to output both an estimate of domestication‐introgression in the focal wild population, as well as an estimate of domestication‐admixture for all individuals in the focal wild population. These estimations were obtained from two approaches: either the exact admixture derived from the individual‐ancestry controlled within the model itself, or from an estimation of admixture using genetic markers in the model coded with varying degrees of selection and influence on the phenotypic traits. Second, we used the model to simulate introgression of domesticated escapees in a wild population using a “realistic” time‐line (up to 50 years) and levels of spawning intrusion (5%–25% escapees) (Diserud et al. 2019; Glover et al. 2019) and gene‐flow (Glover et al. 2013; Karlsson et al. 2016).

### 2.2 Key Attributes of IBSEM and Its Modifications

The eco‐genetic model IBSEM has been described in full detail (Castellani et al. 2015), and its key attributes summarized in subsequent studies where it has been used to investigate phenotypic changes in wild populations subjected to domestication introgression and admixture (Castellani et al. 2018; Sylvester et al. 2019). In short, IBSEM models changes in a range of demographic and key life history traits of a wild salmon population in response to introgression from domesticated escapees. IBSEM includes an underlying genetic architecture based on an additive genetic model with a total of 60 genes coding for the phenotype of individuals in the population during all stages of the life cycle as well as three neutral genetic markers. This also includes diploid inheritance and independent inheritance of genes (markers) that display varying influence over the phenotypic traits. Fitness and phenotypic performance differences between the offspring of domesticated and wild parents are parameterized against extensive data regarding all aspects of this as reviewed (Glover et al. 2017). Adult spawners escaping directly from fish farms (i.e., not the offspring of farmed salmon nor hybrids) are given a reduced spawning success according to sex (30% for females 5% for males) (Fleming et al. 1996, 2000). Thus, it is important to note that for any set level of spawning intrusion, gene‐flow is significantly lower.

### 2.3 Modifications to IBSEM

The original version of IBSEM outputs key biological variables (phenotypic traits and abundance) at the population level. The new key features in IBSEM include scripts to also monitor these and additional parameters at the individual‐fish level across generations. These additional features include (1) tracking individual‐fish ancestry (i.e., genetic origin) throughout all generations in which the model is run, (2) tracking allele frequencies in the 63 genetic markers tagged to control the egg, juvenile, and adult fitness traits (survival, growth etc.) ranging from mild to no effect on the traits, in all individual fish through all generations, (3) tracking phenotypic traits such as weight, length, and age of individual juvenile and adult fish. In turn, these modifications permit outputs that closer align with the type of scientific outputs arising from empirical field studies where individual admixture estimates are compared to individual fish phenotypic parameters (e.g., Bolstad et al. 2017, 2021; Besnier et al. 2022).

In addition to the above improvements, the parameterization of IBSEM, which was originally based on the river Os in western Norway, was changed to model the population of the river Etne, also in western Norway. This was primarily a scaling effect and was done in order to facilitate comparison of model outputs to the river Etne, which is emerging as one of the most extensively studied domestication‐admixed wild Atlantic salmon populations (Besnier et al. 2022, 2024; Harvey et al. 2022; Kaland et al. 2023).

Some of the model parameters, particularly those characterizing juvenile and sea growth and mortality rates, were updated to reflect central scientific data emerging after publication of the original model in 2015 (Glover et al. 2017; Jonsson and Jonsson 2017; Skaala et al. 2019). Finally, the fraction of spawners that survive after reproduction and therefore have an opportunity to spawn for a second time in the river was increased from 0.05 to 0.13, and a weight loss equal to about one‐third of their body mass was introduced for these survivors (Kaland et al. 2023; Persson et al. 2023). Full details of the model are given in Material S1, and the new parameterization of IBSEM is given in Material S2. All coding and scripts for the model have been deposited and are thus openly available (Castellani et al. 2025).

### 2.4 Running IBSEM and Outputting Data

IBSEM was run with the following settings in addition to its default parameters. We set the genetic status of the neighboring wild population, which supplies 5% wild strayers per year to the focal wild population, with the same level of introgression as the focal population itself. This reflects a global introgression scenario where all rivers in a region are equally affected by introgression and gives a greater phenotypic and demographic change in the focal wild population due to a reduced buffering effect via straying from non‐admixed fish (Castellani et al. 2018). Monitoring programs measure the percentage of escapees in wild populations and not the absolute numbers of escapees (Diserud et al. 2019; Glover et al. 2019). Therefore, we varied the proportion of domesticated escapees on the spawning grounds of the focal wild population each year from 5% to 25% using 5% intervals. These are values that strongly overlap with the proportions observed in many wild populations in Norway over several decades. In all scenarios, the wild population was initially let to settle for 25 years without intrusion of farmed escapees. Introgression was then allowed for 50 years, in order to overlap with the current timeline of interaction between domesticated and wild conspecifics in the wild (Glover et al. 2017). Finally, introgression was stopped and a 50‐year period of “recovery” followed (i.e., no further escapees). Each introgression scenario was run using 100 replicates, and the resulting demographic and phenotypic changes in the focal wild population and its individuals were plotted. The demographic parameters of the model were initialized as described in Material S2.

### 2.5 Estimating Admixture

Individual fish ancestry was controlled and tracked within the model itself and exported for all adult fish in the population after all intrusion scenarios and replicates for a period of 100 years using 10‐year intervals. In addition, the genotypes controlled within the model for the 63 biallelic genetic markers ranging from neutral to modest influence over the expression of phenotypic traits and relative survival potential for each fish were outputted for all adult fish in the population after all intrusion scenarios and replicates for all 10‐year intervals. Genotypes from all of these fish were thereafter used to compute individual‐fish admixture in STRUCTURE (Pritchard et al. 2000) using the R‐Package ParalellStructure (Besnier and Glover 2013) and according to the standardized statistical approach to detect unilateral gene flow (Karlsson et al. 2014) that has been implemented to estimate domestication admixture in > 250 salmon rivers in Norway (Karlsson et al. 2016; Diserud et al. 2022). For these computations of admixture on individual fish, multiple‐locus genotypes were grouped into neutral/low selected (21 markers including all three completely neutral markers and the 18 markers displaying the weakest influence over traits and survival probability), highly selected (21 markers displaying the strongest influence over traits and survival probability) and medium selected (21 markers between the two sets described). Thus, the genetic make‐up of each individual fish (and the corresponding population averages) was characterized based on the true ancestry from the model itself, and on estimated admixture from neutral and low to markers under strong selection. These data were paired with individual phenotypic information for the same individual.

## 3 Results

### 3.1 Estimating Introgression Using Genetic Markers

**FIGURE 1:**
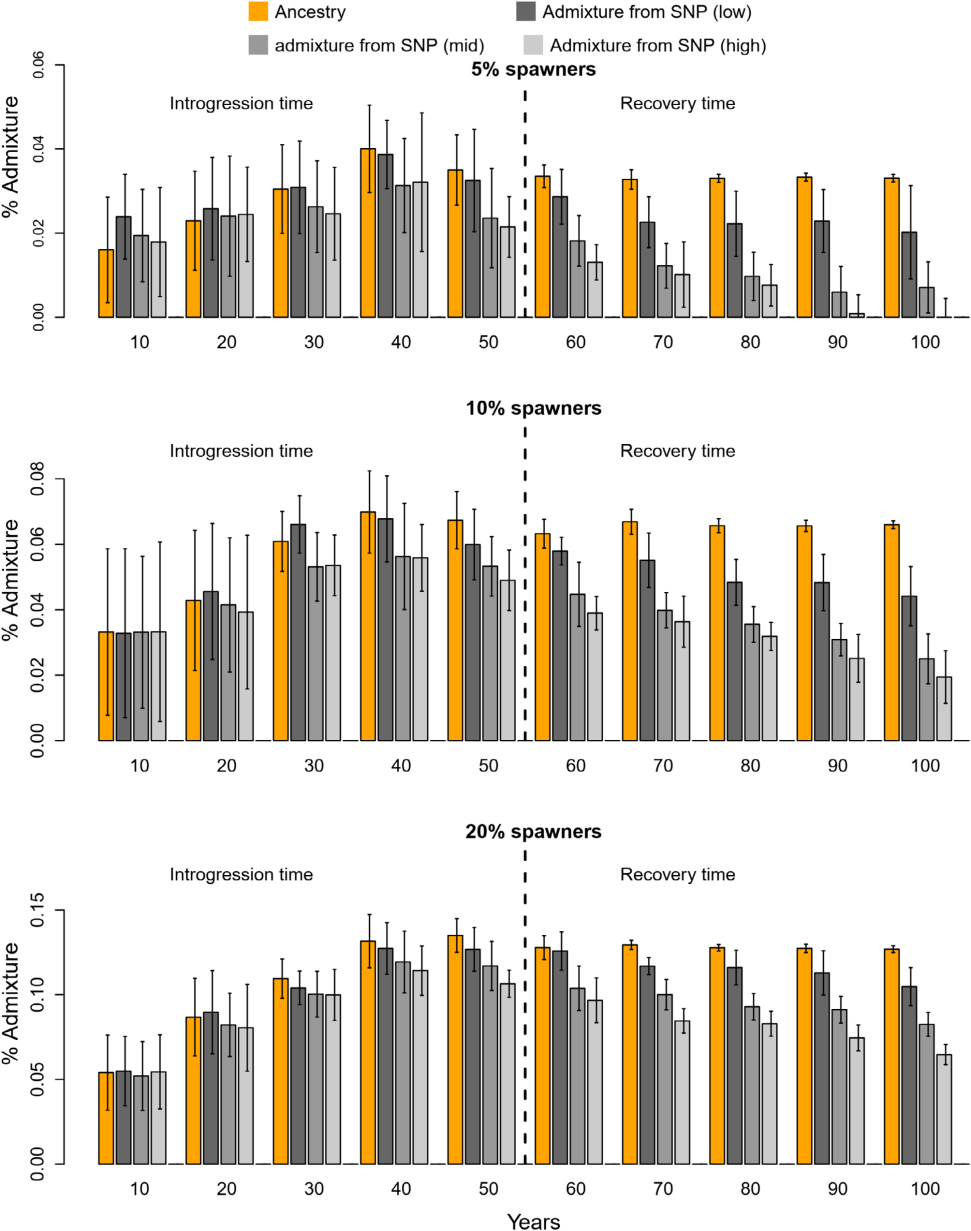
Estimating introgression of domesticated salmon in a wild population using ancestry tracked within the model itself, and with three sets of molecular genetic markers under low to high degrees of selection. Modeling is conducted with 50 years of introgression and then 50 years of “recovery” (i.e., without introgression). Results are shown for 5%, 10%, and 20% spawning intrusion of domesticated salmon in the wild population. Error bars correspond to the standard deviation of population admixture across replicates. Note, spawning intrusion is not equivalent to gene flow as the domesticated spawners are parameterized with lower spawning success as observed in the literature.

Following spawning intrusion, the estimated level of introgression within the population, whether computed by ancestry or by any of the three sets of genetic markers (=neutral/low, medium, or high degree of selection), increased with the proportion of domesticated individuals on the spawning grounds (Figure 1). Likewise, introgression increased in the period between 0 and 50 years of spawning intrusion, under all scenarios, demonstrating a cumulative effect. Therefore, a given level of introgression in the population could be achieved through different combinations in the magnitude and duration of spawning intrusion.

In some of the simulations, similar estimates of introgression were computed by ancestry or the three sets of genetic markers (Figure 1). However, the degree of correspondence varied with time, that is, whether during the introgression or recovery periods, and between marker sets. Two important trends were detected. First, markers under stronger selection displayed lower introgression estimates in the population than markers under weak selection, the latter of which gave similar estimates to the ancestry estimates. Second, differences in introgression estimates among marker groups increased with time, and especially during the recovery period. For example, after 50 years of the 10% intrusion scenario, the introgression estimate as computed by ancestry was approximately 25% higher than the introgression computed with the set of genetic markers under a high degree of selection (Figure 1 and Table 1). After 50 years of “recovery” (year 100, Figure 1 and Table 1), the difference had increased to more than 300%. This effect was caused by a faster drop in the introgression estimated by markers under strong selection, while the ancestry and the estimate of introgression using the genetic markers under weak selection remained relatively stable despite up to 50 years of population recovery. Thus, once introgression has occurred and domestic alleles have dispersed throughout the population, despite a partial recovery in fitness‐related traits and adult abundance (see sections below), estimates of introgression from ancestry and weakly selected markers remained quite stable through time. In contrast, the introgression estimates from markers under strong selection decreased with time, reflecting the purging of domestic alleles associated with negative fitness.

**TABLE 1:** Response of a wild population when modelled with 5%–25% domesticated escapees on the spawning grounds during years 1–50, and then “recovery” in years 51–100.

	Adult spawner intrusion (%)	Years				
		1	25	50	75	100
Abundance adults	**5**	1736	1687	1714	1762	1817
	**10**	1696	1637	1547	1644	1678
	**15**	1722	1573	1536	1689	1634
	**20**	1815	1538	1454	1525	1565
	**25**	1759	1522	1406	1438	1526
Abundance smolts	**5**	106,272	103,461	104,102	101,353	111,079
	**10**	105,377	104,355	100,342	104,389	103,427
	**15**	106,377	98,642	101,044	95,984	95,392
	**20**	108,441	94,619	93,852	87,461	95,087
	**25**	105,451	94,525	85,392	91,051	90,542
Abundance eggs	**5**	6,088,002	6,077,229	6,137,684	6,243,820	6,419,605
	**10**	6,196,939	5,988,729	5,618,166	5,768,656	5,978,491
	**15**	6,437,886	5,977,698	5,733,496	6,042,221	5,887,800
	**20**	6,902,848	6,027,644	5,518,140	5,825,376	5,748,579
	**25**	6,758,562	6,037,646	5,439,105	5,299,761	5,545,675
True ancestry computed within the model itself	**5**	0	0.022	0.034	0.032	0.032
	**10**	0	0.041	0.067	0.066	0.062
	**15**	0	0.071	0.103	0.097	0.093
	**20**	0	0.086	0.133	0.131	0.123
	**25**	0	0.103	0.164	0.158	0.148
Admixture estimated from markers under high selection	**5**	0	0.020	0.020	0.010	0.00
	**10**	0	0.039	0.048	0.036	0.019
	**15**	0	0.063	0.081	0.052	0.038
	**20**	0	0.080	0.106	0.084	0.064
	**25**	0	0.097	0.131	0.121	0.088
Admixture estimated from markers neutral/low selection	**5**	0	0.025	0.023	0.022	0.021
	**10**	0	0.044	0.059	0.055	0.044
	**15**	0	0.070	0.092	0.088	0.073
	**20**	0	0.089	0.126	0.116	0.104
	**25**	0	0.120	0.156	0.148	0.133

*Note:* Population abundance is reported as the number of adult fish returning to the river each year, smolts leaving the river each year, and eggs deposited in the river each year. Ancestry and admixture estimates refer to 0 = pure wild; 1 = pure domesticated. Each data point is the mean of 100 replicates. Note that population abundance upon initiation is different for each scenario (and replicate) due to stochasticity in the model. Full table with 95% confidence intervals presented in File S5.

### 3.2 Estimating Demographic Changes in the Population

**FIGURE 2:**
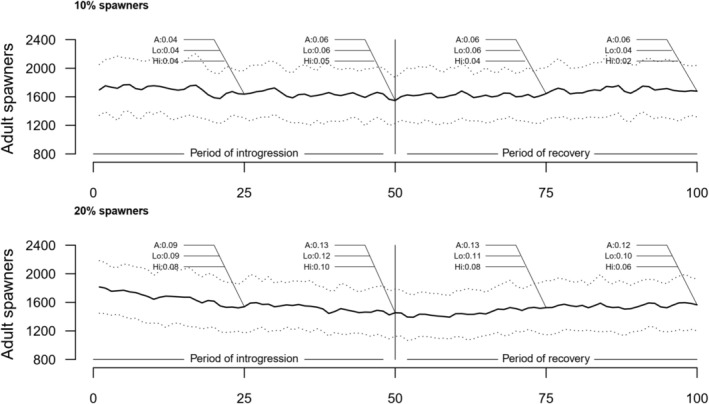
Response of a wild population, measured as adult spawner abundance, in relation to spawning intrusion from 10% (top) 20% (bottom) domesticated escapees during 50 years of introgression then 50 years of “recovery”. Black line represents the mean of 100 runs while stipulated lines above and below reflect the mean ± standard deviation across 100 replicates. Numbers annotated within the figure for every 25 years reflect population admixture estimates at those time‐points as estimated by A, Lo , and Hi. Exact values for population abundance changes including their associated introgression estimates for all intrusion scenarios are found in Table 1 and in the File S5. A, ancestry tracked within the model; Hi, genetic markers under high selection; Lo, genetic markers that are neutral or under weak selection.

Under all but the 5% intrusion scenarios, the number of adult salmon returning to spawn in the wild population declined with time (Figure 2 presenting 10% and 20% escapee scenarios, Table 1 presenting full numerical details for 5%–25% escapee scenarios, Material S3 for statistical results). The observed decrease in the adult population abundance was greater after 50 years as opposed to, for example, 20 years of gene‐flow, and at, for example, 25% as opposed to 5% escapees. Once introgression was stopped at year 50, the population abundance started to recover, although it did not completely attain pre‐introgression levels during the 50‐year recovery period. In regard to the recovery period, it is important to remember that the true ancestry and weak‐selection marker‐based estimates of introgression stayed relatively stable, despite the increasing number of adults returning to the population (compare Figure 1 and Figure 2, or see full details in Table 1). In contrast, the introgression estimate based on the markers under strong selection started to recover to pre‐introgression levels as the population abundance started to recover (compare Figure 1 and Figure 2, or see full details in Table 1).

Population productivity can be measured in multiple ways, but in Atlantic salmon and closely related species, estimated egg deposition via the number of adult females and their total weight is often used to estimate population spawning targets to achieve carrying capacity on a per river basis. Offspring of domesticated and admixed salmon display lower survival rates in the wild but a larger size at age (observed in both empirical and modelled data), and as female size is strongly linked to egg production, the impact on recruitment through the lower number of fish returning in the river may be partially compensated by the increase in their size. Despite this potentially compensating mechanism, results from the model show that although the relative decline in egg production is less than the decline for adult abundance, there is still a decline with introgression (Table 1).

### 3.3 Investigating the Relationship Between Individual‐Fish Admixture and Size at Age

**FIGURE 3:**
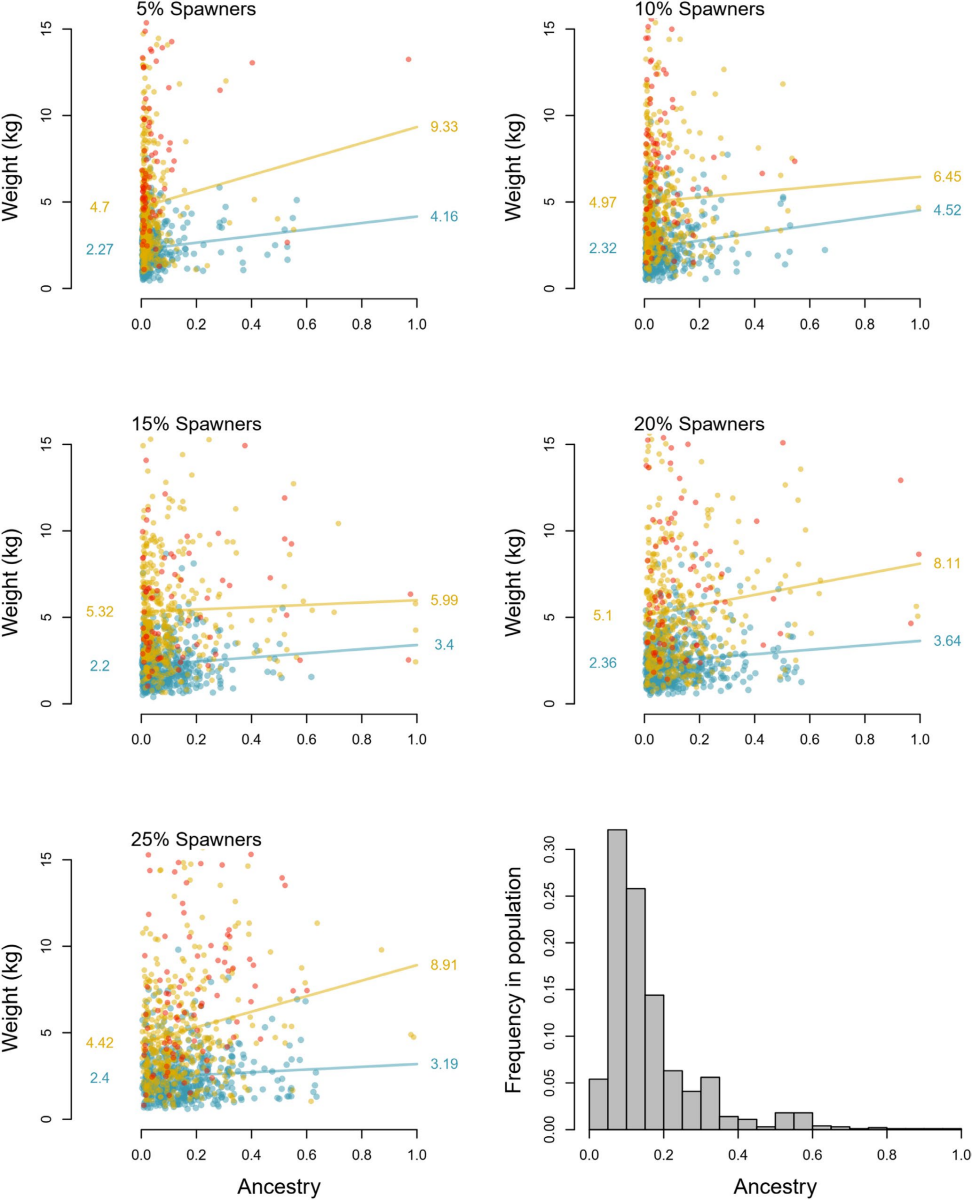
Relationship between individual‐fish admixture as computed using ancestry tracked within the model, and adult weight at age, for the 5%–25% spawning intrusion scenarios. Data presented for 1–3 sea winter salmon (1sw blue bottom line, 2sw yellow top line, 3sw red dots no line due to low N). Figure (bottom right) also shows the distribution of individual‐fish ancestry under the 25% spawning intrusion scenario after 50 years of gene‐flow.

Plotting individual‐fish admixture estimates against their size at age revealed insights into the phenotypic response of the population and how this varies over time and with the types of markers used to compute admixture (see Figure 3 for 50 year scenario based upon ancestry, Material S4 for both 50 and 100 year scenarios for computations based upon ancestry, low or highly selected markers). After 50 years of intrusion, individual admixture was positively correlated with individual fish size (Figure 3). This trend was also observed when individual admixture was computed using weakly selected markers and markers under strong selection (Material S4). Thus, during the introgression phase, there was reasonable concordance in the phenotypic response of individual fish in the population, when admixture was computed by the different sets of markers. After the 50 year “recovery” phase (year 100 in figures in Material S4), however, large differences in these trends became apparent across marker types used to compute admixture. At this time point, ancestry had become non‐informative as all individuals ended up with a similar ancestry. When individual admixture was computed using the set of markers under weak selection, a crash in the strength of the relationship between admixture and individual fish size was observed in comparison with the trend after 50 years of introgression, while for the markers under stronger selection, this change was less evident. Clearly, in the recovery phase, the rapid decoupling of the neutral and weakly selected markers from adult size at age was underpinning this change.

### 3.4 Comparing Model Outputs to Empirical Data From Published Field Studies

**FIGURE 4:**
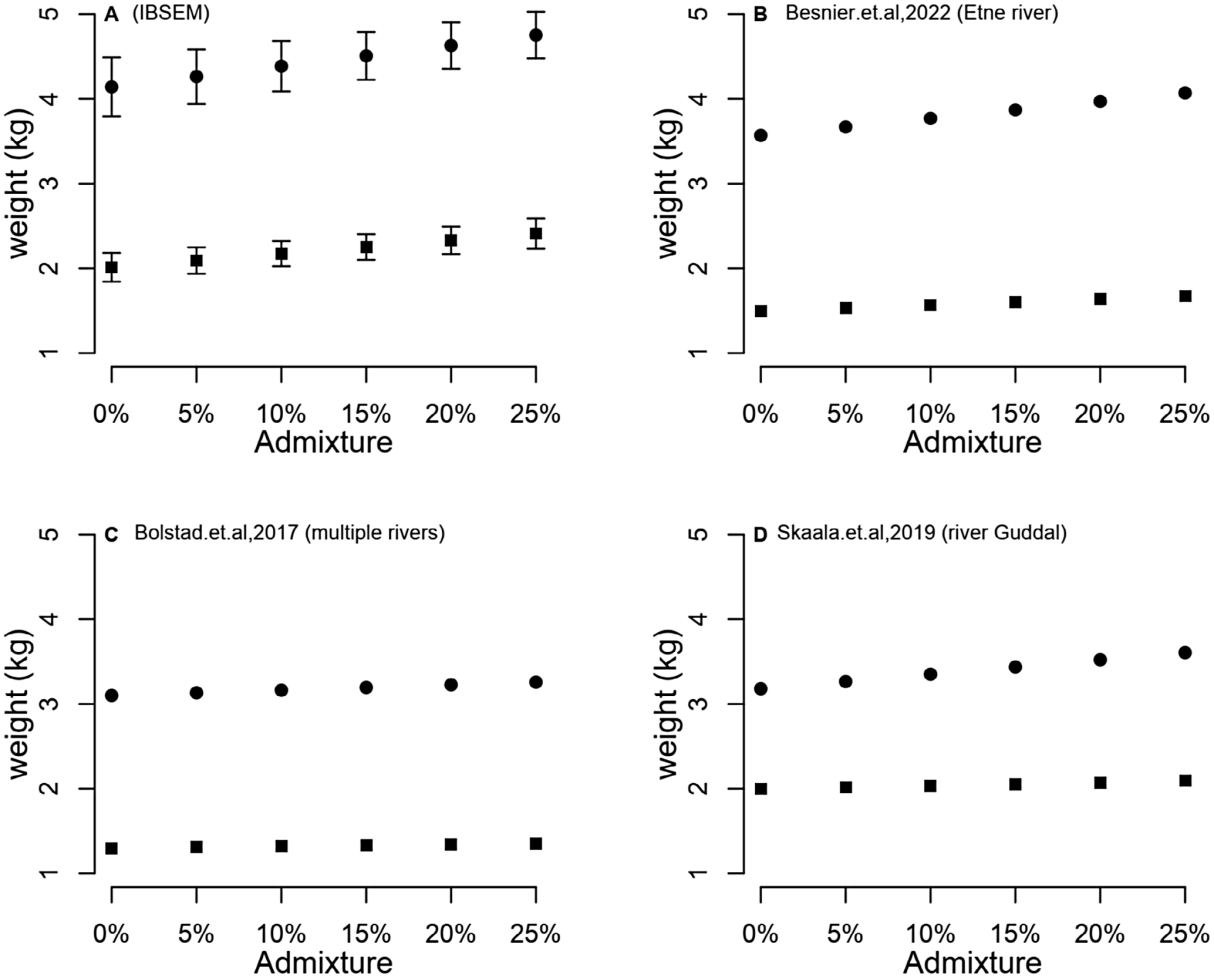
The relationship between mean admixture and mean adult weight for a population displaying 0%–25% introgression for age 1 (filled squares) and age 2 (filled circles) sea winter salmon. Plot (A) (top left) represents data extracted from IBSEM modeling averaged over all runs and spawning intrusion scenarios forming the basis for Figure 3. Plot (B) (top right) is extracted from empirical data from the river Etne which is > 20% admixed (Besnier et al. 2022). Plot (C) (bottom left) is extracted from empirical data averaged over 63 Norwegian salmon populations displaying varying degrees of admixture (Bolstad et al. 2017). Plot (D) (bottom right) is extracted from empirical data originating from planting domesticated, hybrid, and wild salmon in the river Guddal and following growth to adulthood (Skaala et al. 2019).

Using data from all five of the individual‐fish admixture vs. adult size at age regressions from Figure 3, we estimated the mean phenotypic change in fish size at age for the population at 5%–25% introgression using ancestry within the model (Figure 4A). The estimated increase in mean fish size within the population at higher population‐level introgression levels is consistent with the fact that individual fish with higher admixture estimates display higher size at age (Figure 3). The increase in mean fish size within the IBSEM modeled population for a given level of introgression (Figure 4A) was greater than for genetic‐marker based estimates extrapolated directly from field studies including one single wild population (Figure 4B) (Besnier et al. 2022) and from multiple populations in a meta‐analysis (Figure 4C) (Bolstad et al. 2017). Data from the river Guddal where pedigree‐defined families were planted out into the wild showed size‐change estimates closer to the data modeled here (Figure 4D).

## 4 Discussion

As we expand our use of domestication to optimize global food production, it is becoming increasingly important to understand the consequences of interactions between domesticated and wild conspecifics in the natural environment. Here, we expanded the IBSEM model. These changes, for the first time to our knowledge, have enabled the investigation of the phenotypic consequences of domestication introgression at the individual level when computing admixture with markers under varying degrees of selection. Given that genetic markers of unknown neutrality are routinely used to quantify introgression of domesticated genomes in wild Atlantic salmon populations (Wringe et al. 2018; Bolstad et al. 2021; Besnier et al. 2022; Diserud et al. 2022), and in many other species where similar challenges exist, the current study represents an important contribution to help interpret data arising from studies conducted on populations in the wild. Our modeling also provides new insights into fitness consequences of introgression, which in turn can assist the authorities in their regulation of the environmental footprint of Atlantic salmon aquaculture (Taranger et al. 2015; Glover et al. 2019, 2020). Finally, the comparison of IBSEM's domestication‐driven phenotypic changes against the best available field data represents a rare validation of a model of this sort.

### 4.1 Estimating Introgression Using Markers Under Varying Degrees of Selection

Panels of “diagnostic” genetic markers to identify domesticated, wild, and admixed Atlantic salmon in Norway (Karlsson et al. 2011; Besnier et al. 2022) and North America (Liu et al. 2017; Wringe et al. 2019; Nugent et al. 2023) have been mined from genomic resources. In turn, these panels of markers have been used to quantify introgression and its biological consequences in wild Atlantic salmon populations (Glover et al. 2013; Karlsson et al. 2016; Bolstad et al. 2017; Wringe et al. 2018; Besnier et al. 2022). Although such work is well‐developed in Atlantic salmon, it is also important in other plant and animal systems where genetic markers are used to quantify the effects of domestication–introgression in native populations. Therefore, there is a generic need to understand the consequences of using such markers to estimate introgression and admixture, and thereafter, how this may influence our understanding of the biological consequences.

There is a general literature addressing the challenges of measuring introgression and admixture using genetic markers under varying degrees of selection (Ellstrand et al. 1999; Edmands 2007; Huisman and Tufto 2013), and many factors play a role. For example, the number and genomic distribution of markers, the strength of selection, recombination rates, effective population sizes, and the duration and magnitude of gene flow. However, previous knowledge in addition to the modeling here (Figure 1) all show that genetic markers under stronger selection give different estimates of introgression and admixture than markers that are neutral or under weak selection. This needs to be kept in mind when interpreting introgression and admixture estimates in wild populations.

Our modeling showed that during the early period of gene flow, under the continued input of domesticated escapees into the target population, differences in admixture estimates between sets of markers were low to modest (Figure 1). This is due to the continued input of domesticated alleles compensating for the purging of domesticated alleles in markers under selection. Therefore, all the markers are still providing information regarding the introgression history on this timeline. However, and importantly, we detected an increasingly divergent admixture estimate between sets of markers when looking in the “recovery” period. Once gene flow was stopped in the model, and as natural selection pushed phenotypic traits and fitness back towards their pre‐impact optima, markers responsible for these traits also reverted in tandem. Inversely, neutral and weakly selected markers for which allelic frequencies are primarily affected by gene flow and drift in the model (and additionally by hitch‐hiking selection in the real world), gave largely unchanged admixture estimates once they had been dispersed within the wild population itself (Figure 1). This work thus illustrates how fitness‐related traits and their underlying genes start to recover once introgression has ceased and natural selection plays the dominating evolutionary force, and simultaneously, how neutral and close to neutral markers persist in the population for a long period of time thus reflecting the historical footprint of introgression.

The observations discussed immediately above may help us interpret results from an earlier modelling study which suggested an almost complete feralization of an Atlantic salmon population in a 100 year period following just 12 years of gene‐flow from domesticated conspecifics under specific modelling conditions (Hindar et al. 2006). The work in question used empirically relevant fitness differentials between wild and domesticated fish. However, it was constructed as an “ancestry type model” without inferences on phenotypic or fitness effects on the population as is the case in IBSEM. Results from the model by Hindar and colleagues align with the results from IBSEM for the markers that are neutral or under only weak selection, during the recovery period. That is, a population can undergo a fitness/phenotype recovery while still displaying heavily swamped domestication. It therefore follows that one needs to show caution in inferring biological changes in wild populations based upon admixture estimates from markers without considering the degree of selection and the time‐line of intrusion and/or recovery. This is especially so as multiple‐generation experiments in the field (McGinnity et al. 2003) and from the modelling here and earlier (Castellani et al. 2015, 2018), all suggest a gradual return towards pre‐impact fitness once gene‐flow has ceased.

In light of the above discussion, an important question that still remains unanswered through empirical work is “to what degree are genetic marker panels currently being implemented to quantify introgression and its effects in wild populations, under selection”? In order to answer this, further studies of selection at the molecular level in the wild (Besnier et al. 2015; Wacker et al. 2021), also across generations, are needed. Nevertheless, while awaiting empirical data on this matter, we can still draw insights from current knowledge. Studies of domesticated Atlantic salmon have identified numerous genomic regions under selection (Lopez, Benestan, et al. 2019; Lopez, Linderoth, et al. 2019; Naval‐Sanchez et al. 2020). However, the underlying functional genomic differences between wild and domesticated salmon are highly polygenic, with no evidence of genes of major influence (Karlsson et al. 2011; Bicskei et al. 2014; Besnier et al. 2020, 2022; Naval‐Sanchez et al. 2020). Furthermore, the signatures of domestication selection only display weak evidence of parallelism among the different domesticated strains (Lopez, Benestan, et al. 2019; Lopez, Linderoth, et al. 2019; Naval‐Sanchez et al. 2020), reflecting a high degree of genetic heterogeneity among domesticated strains. Therefore, while some of the markers being used in farmed vs. wild panels as of today (Karlsson et al. 2011; Wringe et al. 2019; Besnier et al. 2022) are likely to be under some degree of selection, they are unlikely to display the same level of influence over the fitness traits as the high selected marker panel in the present study. For example, the marker displaying the strongest fitness and phenotypic influence in IBSEM has a 15% effect over the trait(s), which is higher than any known gene differentiating between domesticated versus wild Atlantic salmon. Therefore, in light of the above, together with the fact that domestication signatures are highly polymorphic and largely non‐parallel across the multiple strains used in aquaculture, we suggest that it is most likely that domesticated vs. wild marker panels currently in use are likely to primarily behave as neutral to under weak selection. We nevertheless acknowledge that this is possibly context specific, will vary from panel to panel, and that specific investigation is needed to address this for each panel in use.

### 4.2 Phenotypic Impacts on the Population

Studies of wild Atlantic salmon populations subjected to spawning intrusion from domesticated conspecifics have demonstrated a relationship between individual‐fish admixture, as computed by panels of genetic markers, and phenotypic (Bolstad et al. 2017; Besnier et al. 2022) or phenological traits (Besnier et al. 2022). Earlier work with IBSEM suggested that at up to ~10% escapees on the spawning grounds, equating to ~2% gene flow/year in the model due to parameterizing for the documented lower spawning success of farmed escapees (Fleming et al. 1996, 2000), causes only marginal changes in life‐history traits at the population‐average level (Castellani et al. 2015, 2018). The updated version of IBSEM presented here now provides an output of individual fish data (Figure 3), the outputs of which are highly consistent with results from studies in the field (Bolstad et al. 2017, 2021; Besnier et al. 2022) (Figure 4). Specifically, at ~5%–10% introgression levels, which requires ~10%–25% escapees on the spawning grounds for ~20–50 years to achieve in IBSEM (Figure 1 vs. Figure 4 or Table 1), the population‐average phenotypic changes in size at age were small and closely align with data from both (Besnier et al. 2022) and (Bolstad et al. 2017) (Figure 4). Thus, data from the most advanced empirical studies conducted in the field, in addition to the modeling work presented here, all point in the direction of limited phenotypic changes in wild populations at spawning intrusions and timelines that achieve less than 10% introgression. Changes at higher levels of introgression are also relatively modest, although the model and empirical evidence clearly show a scaling of spawning intrusion and the resulting level of phenotypic change. The above is consistent with an earlier empirical study demonstrating that the combined influence of phenotypic plasticity and selection contributes to the modest observed phenotypic differences between domesticated and wild fish in the natural environment (Glover et al. 2018) while they are more clearly different when communally reared in the domesticated environment with high food rations and no selection (Solberg et al. 2013; Harvey et al. 2016).

### 4.3 Effects on Population Abundance?

Our modeling here, together with earlier modeling work, shows that a decline in adult population abundance may occur in wild populations resulting from introgression of domesticated conspecifics. While we observed a partial compensation in egg deposition via the higher size at age of admixed fish, as has been observed and/or implied in the wild (McGinnity et al. 2003; Skaala et al. 2019), a reduction in population size in both adults and deposited eggs was noticed in the model here, scaling with the level of spawning intrusion (Table 1 and Figure 2). However, as for phenotypic changes, changes in abundance were low at low intrusion rates.

In comparison with wild conspecifics, the offspring of domesticated, F1 hybrid, and backcrossed Atlantic salmon display lower freshwater survival (McGinnity et al. 1997, 2003; Fleming et al. 2000; Skaala et al. 2012, 2019). Within‐cohort studies conducted in domestication‐admixed wild populations have also reported a reduction in admixture during the freshwater stage, confirming selection against domesticated and/or admixed individuals (Sylvester et al. 2019; Wacker et al. 2021). The available empirical evidence is thus consistent with a significant genetic‐based fitness difference during this stage of the life cycle. However, and in contrast, empirical evidence for survival differences during the marine stage of the life cycle is both less abundant and inconsistent. While one study has shown reduced relative marine survival of domesticated and F1 hybrid salmon that were hatched in the wild (Skaala et al. 2019), another did not observe such differences (Fleming et al. 2000). Release studies using hatchery‐produced smolts of domesticated, F1 hybrid, and wild genetic background have shown lower survival of fish of domesticated background in the marine phase (McGinnity et al. 2003; Jonsson and Jonsson 2017; Skaala et al. 2019). However, those releases involve fish that have not been exposed to natural selection during the freshwater phase, and results must be interpreted with that fact in mind. In addition, those releases have identified maternal and/or non‐additive inheritance patterns (Jonsson and Jonsson 2017; Skaala et al. 2019) that complicate the picture of marine survival differences further. Therefore, while IBSEM is coded with reduced survival probabilities for domesticated and admixed salmon for both the freshwater and marine stages of the life cycle, which contributes to the low but significant decline in adult abundance following introgression in the model, empirical evidence for the marine stage of the life cycle is conflicting. Consequently, we suggest that IBSEMs predictions on population abundance may overestimate the true demographic cost of introgression and need to be interpreted with caution. Further empirical data on this critical stage of the life cycle is clearly needed.

### 4.4 Potential Caveats of the Modelling Work

In addition to the uncertainty raised above, that is, what are the differences in marine survival between domesticated vs. wild offspring in natural populations, there is also the question of whether the observed lower relative freshwater survival of domesticated offspring in the wild leads to reduced freshwater production or not, due to the influence of density‐dependent selection in this life stage (Bult et al. 1999; Armstrong and Griffiths 2001). There are also further limitations in the modeling work that display potential to influence the result. First, epigenetic effects may well influence the process of domestication (Podgorniak et al. 2022; Koch et al. 2023), although the degree to which this plays a role in the survival of domesticated offspring in the natural environment still remains unquantified. Second, although the model is coded with relative spawning success of 30% and 5% for female and male escapees entering the river respectively (Fleming et al. 1996, 2000), these estimates are old and thus in need of updating. It is also likely that the spawning success of domesticated escapees varies greatly in time and space and will, among other things, depend on how long escapees have been in the wild prior to entering a river to spawn (Strand et al. 2024) as prior experience in the wild is likely to increase spawning success (Fleming et al. 1997). Third, the underlying genomic differences between domesticated and wild Atlantic salmon are still largely unknown, and the architecture chosen in IBSEM, with 63 genes displaying varying degrees over the trait, is likely to reflect a simplification. Clearly, future work will improve our knowledge of this situation and thereafter improve models. Nevertheless, it is important to reiterate that the phenotypic response predictions from IBSEM strongly align with empirical data from the field.

### 4.5 Management Implications

Policy makers require knowledge to establish management‐thresholds that permit balancing the needs of an aquaculture industry against its potential negative impact(s) on the environment. In Norway, which is the world's largest producer of farmed Atlantic salmon, the national monitoring program reports the proportions of domesticated escapees observed in > 200 rivers annually (Glover et al. 2019). This uses the categories 0%–4%, 4%–10%, and > 10% escapees on the spawning grounds to reflect a low, moderate and high risk of genetic change (Taranger et al. 2012). In parallel, genetic‐marker based domestication‐introgression estimates also exist for > 250 Norwegian salmon populations, using a four‐category system whereby > 10% introgression is characterized as “very large genetic changes documented” (Diserud et al. 2022). Work in Canada using the IBSEM model on empirical data from that region uses 10% escapees on the spawning grounds as the threshold for genetic impact (Bradbury et al. 2020). The Norwegian data have been used together with other relevant information in an annual risk assessment that projects the likelihood for further introgression of domesticated salmon escapees in wild populations within rivers in each of Norway's 13 aquaculture production zones (Glover et al. 2020). This Norwegian assessment, and global management guidelines for Atlantic salmon in general, are built on the broadly‐accepted principles that introgression from domesticated conspecifics may lead to losses of local adaptation, elicit demographic and phenotypic consequences, and in the long‐term, challenge the evolutionary trajectory of affected populations (Hindar et al. 2006; Laikre et al. 2010; Glover et al. 2017).

With respect to the management thresholds and risk assessments detailed above, the modeling work here provides two novel and important insights that may assist future adjustments within and outside Norway. First, while it is beyond the scope of this work to suggest new management boundaries, our results indicate that management thresholds currently in use will offer a high level of protection for native populations. Or put alternatively, within the time‐frame studied, none of the results from our modeling work, nor relevant data from field studies or earlier modeling exercises, indicate more than small effects on native populations when the proportion of escapees on the spawning grounds is less than 10% and/or cumulative introgression is less than 10%. Second, our work clearly illustrates that molecular‐marker based introgression estimates in wild populations need to be critically interpreted, given the disparity revealed here between those estimates generated with genetic markers displaying different levels of neutrality, and that admixture estimates based upon markers that were either neutral and/or under only weak selection remained in the population despite a partial recovery in the fitness traits. Introgression in many Norwegian Atlantic salmon populations has occurred over a period of more than three decades, and the frequency of escapees entering rivers has been declining (Diserud et al. 2019; Glover et al. 2019). Thus, it is possible that the molecular marker generated admixture estimates for Norwegian populations more accurately reflect the history of introgression than the current fitness consequences.

## Conflicts of Interest

The authors declare no conflicts of interest.

## Supporting information


**Appendix S1:** eva70140‐sup‐0001‐AppendixS1.docx.

**Appendix S2:** eva70140‐sup‐0002‐AppendixS2.docx.

**Appendix S3:** eva70140‐sup‐0003‐AppendixS3.xlsx.

**Appendix S4:** eva70140‐sup‐0004‐AppendixS4.docx.

**Appendix S5:** eva70140‐sup‐0005‐AppendixS5.xlsx.

## Data Availability

There are no raw data associated with this work.
