# Supplementary material for: Modelling the Consequences of Domestication‐Introgression in Wild Populations Using Genetic Markers Under Varying Degrees of Selection
Source: Evol Appl. 2025 Sep 19;18(9):e70140. doi: 10.1111/eva.70140 (PMC12446728; doi:10.1111/eva.70140)
Supplement: Supplementary file 2 — Appendix S2: eva70140‐sup‐0002‐AppendixS2.docx. [file EVA-18-e70140-s002.docx]

**IBSEM: AN INDIVIDUAL-BASED ATLANTIC SALMON POPULATION MODEL**

# K. A. Glover^1*^ M. Castellani^2^, F. Besnier^1^, M. Heino^1, 3^

# *1 = Institute of Marine Research, Bergen, Norway*

# *2= Department of Mechanical Engineering, University of Birmingham, UK.*

# *3 = Department of Biological Sciences, University of Bergen, Bergen, Norway*

* Corresponding author: E-mail: kevin.glover@hi.no

# APPENDIX S2

# PARAMETERISATION OF MODEL

B.1 PHYSICAL ENVIRONMENT

**The river area for the freshwater phase was modelled on that of the Etne river in Innlandet county, Norway.** For the oceanic phase, the temperatures reproduce the monthly average sea surface temperatures (SSTs) measured in the 30 years period 1982-2012 in the Norwegian Sea (NOAA_ERSST_V3 data provided by the NOAA/OAR/ESRL PSD, Boulder, Colorado, USA, from their Web site at <http://www.esrl.noaa.gov/psd/>).

Table A gives the standard values of the environmental parameters.

| **Freshwater stage** | | **Oceanic stage** | |
| --- | --- | --- | --- |
| **River area=1000,000 m^2^** | | **-** | |
| **Average water temperatures (^º^C)** | | | |
| Month | River | | Sea |
| January | 2.00 | | 4.82 |
| February | 2.00 | | 4.65 |
| March | 2.00 | | 4.67 |
| April | 5.00 | | 5.16 |
| May | 11.00 | | 6.3 |
| June | 16.00 | | 7.91 |
| July | 18.00 | | 9.15 |
| August | 18.00 | | 9.3 |
| September | 14.00 | | 8.26 |
| October | 9.00 | | 6.96 |
| November | 5.00 | | 5.89 |
| December | 3.00 | | 5.23 |
| Standard deviation $\sigma_{T}$ on monthly temperatures | 1.0 | | 0.3 |

**Table A** Environmental parameters.

B.2 DEMOGRAPHY

The Individual-Based Salmon Eco-genetic Model (IBSEM) reproduces the life cycle of an Atlantic salmon (Salmo salar L.) population. The model divides the life history of the individuals into three main phases: embryonic (egg to the end of endogenous feeding on its embryonic yolk-sac reserves, E), freshwater (juvenile, J), and oceanic (adult, A).

The equations described in S1 File have been parameterized to reproduce the demographics Atlantic salmon in the river Etne in Norway. The following tables list the settings of the parameters.

*B.2.1 Growth*

| **Parameter** | **Phase** | ***X* (Age)** | **Value** | **Measurement unit** |
| --- | --- | --- | --- | --- |
| $A\left( X \right)$ | *J* | *p0* | 0.75 | g/days |
|  |  | *p1* | 0.7 | g/days |
|  |  | *p2* | 0.5 | g/days |
|  |  | *sm* (young-of-the-year) | 0.7 | g/days |
|  |  | *sm* (older) | 0.6 | g/days |
|  | *A* | *0SW* | **4.3** | g/days |
|  |  | *1SW*, *2SW*, *3SW* | **1.8** | g/days |
| $b\left( X \right)$ | *J*, *A* | *p0*, *p1*, *p2*, *sm* | 0.31 | - |
| $d\left( X \right)$ | *J* | *p0*, *p1*, *p2*, *sm* | 0.374 | 1/^º^C |
|  | *A* | *0SW*, *1SW*, *2SW*, *3SW* | 0.33 | 1/^º^C |
| *g*$\left( X \right)$ | *J* | *p0*, *p1*, *p2*, *sm* | 0.201 | 1/^º^C |
|  | *A* | *0SW*, *1SW*, *2SW*, *3SW* | 0.2 | 1/^º^C |
| $s_{1}$ | *E* | *eg* | 1.6345 | - |
| $s_{2}$ | *E* | *eg* | 12.991 | mm |
| $T_{L}\left( X \right)$ | *J* | *p0*, *p1*, *p2* | 7 | ^º^C |
|  |  | *sm* | 0 | ^º^C |
|  | *A* | *0SW*, *1SW*, *2SW*, *3SW* | 2 | ^º^C |
| $T_{U}\left( X \right)$ | *J* | *p0*, *p1*, *p2* | 24 | ^º^C |
|  |  | *sm* | 24 | ^º^C |
|  | *A* | *0SW*, *1SW*, *2SW*, *3SW* | 20 | ^º^C |
| $\beta_{dens}(X)$ | *J* | *p0* | 20 | - |
|  |  | *p1*, *p2* | 70 | - |
| $\delta_{f}^{A}\left( X \right)$ | *J* | *p0*, *p1*, *p2* | **1.2** | - |
|  |  | *sm* | **1.9** | - |
|  | *A* | *0SW*, *1SW*, *2SW*, *3SW* | **1.15** | - |
| $\delta_{w}^{A}\left( X \right)$ | *all* | *all* | 1 | - |
| $\sigma\left( X \right)$ | *E* | *al* | 0.035 | - |
|  | *J* | *p0* | 0.25 | - |
|  |  | *p1*, *p2* | 0.08 | - |
|  |  | *sm* | 0.09 | - |
|  | *A* | *0SW*, *1SW*, *2SW*, *3SW* | 0.12 | - |

**Table B** Growth parameters setting.

*B.2.2 Mortality*

| **Parameter** | **Phase** | ***X* (Age)** | **Value** | **Measurement unit** |
| --- | --- | --- | --- | --- |
| $dsp\left( X,s \right)$ | J | *p0*, *s*=*warm* | 0.983 | - |
|  |  | *p0*, *s*=*cold* | 0.9988 | - |
|  |  | *p1*, *p2*, *s*=*warm* | 0.9986 | - |
|  |  | *p1*, *p2*, *s*=*cold* | 0.999 | - |
|  |  | *sm* | 0.999 | - |
| $k_{1}$ | A | *0SW*, *1SW*, *2SW*, *3SW* | **925** | 1/mm |
| $k_{2}$ | A | *0SW*, *1SW*, *2SW*, *3SW* | -1.55 | - |
| *m* | E | eg | -3.16 | 1/g |
| q | E | *eg* | 1.12 | - |
| $\alpha(X)$ | E | *eg* | 0.62 | - |
|  | J | *p0* | 0.28 | - |
|  |  | *p1*, *p2*, | 0.3 | - |
| $\beta(X)$ | E | *eg* | 0.26 | m^2^/egg |
|  | J | *p0* | 830 | - |
|  |  | *p1*, *p2*, | 280 | - |
| $\delta_{f}^{S}\left( X \right)$ | E | eg | 0.8 | - |
|  | J | *p0* | **0.93** | - |
|  | J | *p1*, *p2* | **0.96** | - |
|  | J | *sm* | **0.965** | - |
|  | A | *0SW*, *1SW*, *2SW*, *3SW* | **0.95** | - |
| $\delta_{w}^{S}\left( X \right)$ | *all* | *all* | 1 | - |

**Table C** Mortality parameters setting.

*B.2.3 Maturation and Smolting*

| **Maturation** | | | |  |
| --- | --- | --- | --- | --- |
| **Parameter** | **Phase** | ***X* (Age)** | **Value** | **Measurement unit** |
| $K_{1}$ | J | *p0*, *p1 p2* | 0.13 | 1/mm |
| $K_{2}$ | J | *p0*, *p1 p2* | 107 | mm |
| $P\left( X \right)$ | A | *0SW* | 0 | - |
|  |  | *1SW* | 0.4 | - |
|  |  | *2SW* | 0.85 | - |
|  |  | *3SW* | 1 | - |
| $\delta_{f}^{m}\left( X \right)$ | A | *0SW* | 0 | - |
|  |  | *1SW* | 0.25 | - |
|  |  | *2SW* | 0.47 | - |
|  |  | *3SW* | 1 | - |
| $\delta_{w}^{m}\left( X \right)$ | *all* | *all* | 1 | - |
| **Smolting** | | | |  |
| **Parameter** | **Phase** | ***X* (Age)** | **Value** |  |
| $K_{1}$ | J | *p0*, *p1 p2* | 0.2 | 1/mm |
| $K_{2}$ | J | *p0*, *p1 p2* | 103 | mm |
| $\tau$ | J | *p0*, *p1 p2* | 90 | mm |

**Table D** Maturation and smolting parameters setting.


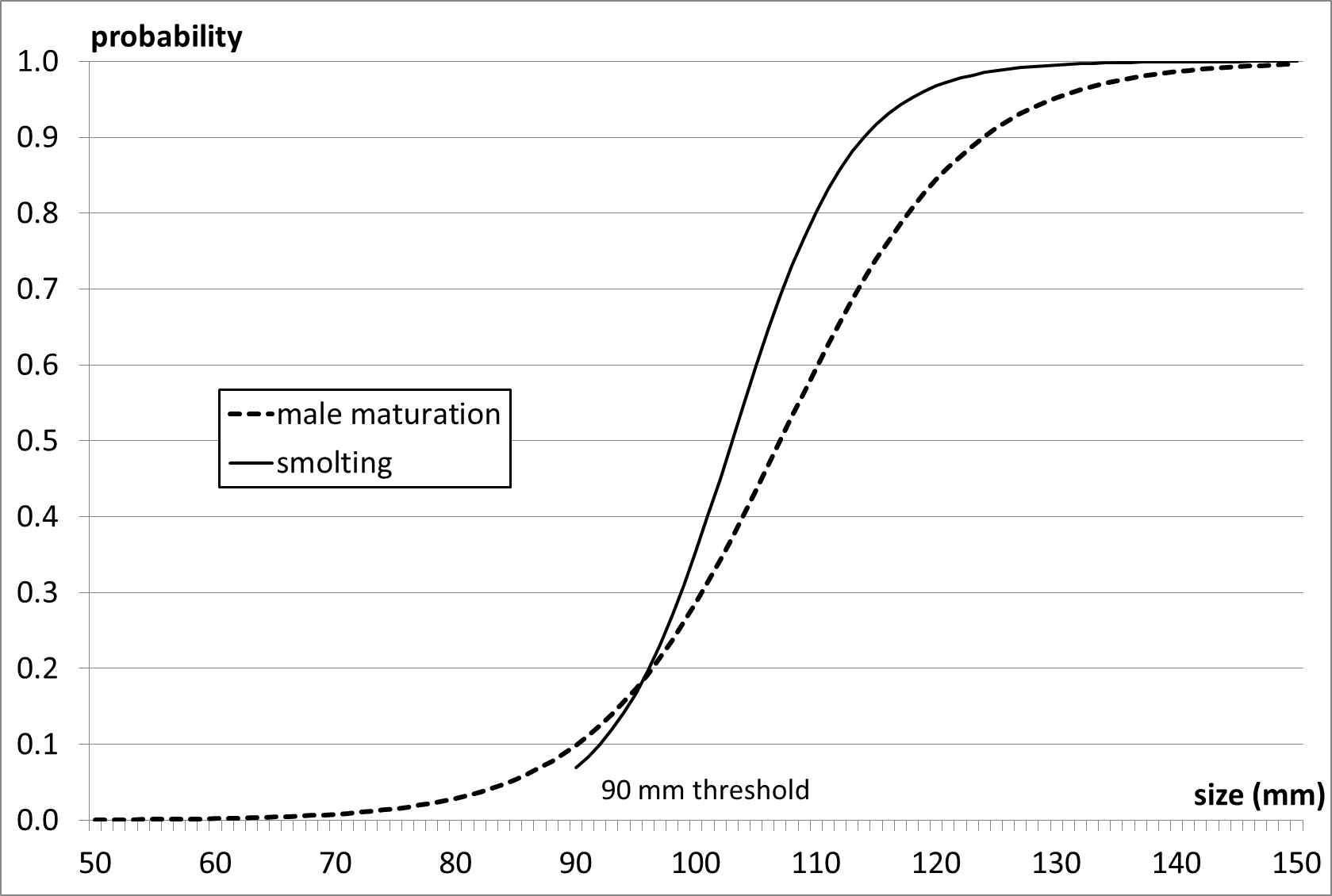


**Fig A.** Fork length-dependent male parr maturation and smolting probabilities. Equations and parameters as described in Tables D in S1 File and D.

*B.2.4 Reproduction and Straying*

| **Parameter** | **Phase** | ***X* (Age)** | **Value** | **Measurement unit** |
| --- | --- | --- | --- | --- |
| $c_{1}$ | A | *1SW*, *2SW*, *3SW* | 0.86 | 1/g |
| $c_{2}$ | A | *1SW*, *2SW*, *3SW* | 1.63 | - |
| $c_{3}$ | A | *1SW*, *2SW*, *3SW* | 0.166 | 1/g |
| $c_{4}$ | A | *1SW*, *2SW*, *3SW* | 5.68 | - |
| $\boldsymbol{f}$ | **A** | ***1SW*, *2SW*, *3SW*** | **0.3** | **-** |
| $m_{min}$ | J | *p0*, *p1 p2* | 0.1 | - |
| $m_{max}$ | J | *p0*, *p1 p2* | 0.3 | - |
| $m_{post}$ | A | *1SW*, *2SW*, *3SW* | **0.87** | - |
| *rs*(*sex*) | A | *1SW*, *2SW*, *3SW* | *sex=male 0.05 sex=female 0.3* | - |
| σ | A | *1SW*, *2SW*, *3SW* | 0.05 | - |
| $\sigma_{NE}$ | A | *1SW*, *2SW*, *3SW* | 0.1 | - |
| $\sigma_{WE}$ | A | *1SW*, *2SW*, *3SW* | 0.02 | - |
| *χ* | A | *1SW*, *2SW*, *3SW* | **same as focal population** | - |

**Table E** Reproduction and strayers parameters setting.

B.3 SOFTWARE IMPLEMENTATION

**The IBSEM model is implemented in C++. With the above parameters, on a machine using an AMD Ryzen 7 6800H processor with 16GB of memory and using Windows 11 Home 64-bit operating system., the execution of one simulation (50 years introgression + 50 years recovery time) takes 53 minutes circa. This time includes several routines currently used to monitor and save population statistics and periodic snapshots of the entire smolt and adult population.**
