# Supplementary material for: Modelling the Consequences of Domestication‐Introgression in Wild Populations Using Genetic Markers Under Varying Degrees of Selection
Source: Evol Appl. 2025 Sep 19;18(9):e70140. doi: 10.1111/eva.70140 (PMC12446728; doi:10.1111/eva.70140)
Supplement: Supplementary file 4 — Appendix S4: eva70140‐sup‐0004‐AppendixS4.docx. [file EVA-18-e70140-s001.docx]

Supplementary material S3

5-25% intrusion for 50 years. Admixture computed bv ancestry – identical to Fig 3. In main text.

5-25% intrusion for 50 years then 50 years of recovery. Admixture computed bv ancestry.

5-25% intrusion for 50 years. Admixture computed by neutral markers

5-25% intrusion for 50 years then 50 years of recovery. Admixture computed bv neutral markers.

5-25% intrusion for 50 years. Admixture computed bv markers under strong selection.

5-25% intrusion for 50 years then 50 years of recovery. Admixture computed bv markers under strong selection.
